# The H3.3K27M oncohistone antagonizes reprogramming in Drosophila

**DOI:** 10.1371/journal.pgen.1009225

**Published:** 2021-07-19

**Authors:** Kami Ahmad, Steven Henikoff

**Affiliations:** 1 Basic Sciences Division, Fred Hutchinson Cancer Research Center, Seattle, Washington, United States of America; 2 Howard Hughes Medical Institute, Seattle, Washington, United States of America; Geisel School of Medicine at Dartmouth, UNITED STATES

## Abstract

Development proceeds by the activation of genes by transcription factors and the inactivation of others by chromatin-mediated gene silencing. In certain cases development can be reversed or redirected by mis-expression of master regulator transcription factors. This must involve the activation of previously silenced genes, and such developmental aberrations are thought to underlie a variety of cancers. Here, we express the wing-specific Vestigial master regulator to reprogram the developing eye, and test the role of silencing in reprogramming using an H3.3K27M oncohistone mutation that dominantly inhibits histone H3K27 trimethylation. We find that production of the oncohistone blocks eye-to-wing reprogramming. CUT&Tag chromatin profiling of mutant tissues shows that H3K27me3 of domains is generally reduced upon oncohistone production, suggesting that a previous developmental program must be silenced for effective transformation. Strikingly, Vg and H3.3K27M synergize to stimulate overgrowth of eye tissue, a phenotype that resembles that of mutations in Polycomb silencing components. Transcriptome profiling of elongating RNA Polymerase II implicates the mis-regulation of signaling factors in overgrowth. Our results demonstrate that growth dysregulation can result from the simple combination of crippled silencing and transcription factor mis-expression, an effect that may explain the origins of oncohistone-bearing cancers.

## Author summary

The differentiation of cell fates in multicellular organisms requires that certain genes be activated, and genes for alternative cell fates are repressed by chromatin silencing. Specific histone mutations that cripple silencing have been found associated with brain cancers in human patients, and these cancers may originate from instability of cell fates. We tested this idea by expressing a wing specification factor in the Drosophila eye to reprogram cell fates and create winged eyes. To test if defects in chromatin silencing increased cell reprogramming, we simultaneously expressed a crippling mutant histone. Contrary to expectations, we found that wing-to-eye reprogramming no longer occurs and instead the eye overgrows, a phenotype reminiscent of the cancers where the histone mutation was first identified. We suggest that reprogramming requires chromatin silencing of the previous developmental program, and that blocking reprogramming can uncouple growth-promoting effects from developmental one of tissue specification transcription factors.

## Introduction

Developmental programs in multicellular organisms are specified by transcription factors that activate and repress batteries of genes, thereby determining cell fate. Ectopic expression of specific transcription factors can drive changes in cell fate, either by inducing pluripotency from a differentiated state [1], or by transforming one cell type to another in a process referred to as ‘transdetermination’ or as ‘direct reprogramming’ [2]. Aberrant reprogramming induced by transcription factor misexpression underlies some developmental and malignant diseases.

In eukaryotes transcription factors interact with chromatin, where genomic DNA is wrapped around histone octamers in nucleosomes. Silencing histone modifications on nucleosomes inhibit factor binding and transcription, and so modulate gene expression programs. Silencing has been suggested to impose directionality and reliability to developmental progression [3]. A key chromatin mechanism is mediated by trimethylation of the lysine-27 residue of histone H3 (H3K27me3) which is bound by Polycomb proteins. Mutations of Polycomb proteins derepress developmental transcription factor genes and thereby induce aberrant fate transformations in animals, plants, and fungi, highlighting the conserved importance of this chromatin system [4–6].

In Drosophila, mutation of the histone H3-K27 residue recapitulates Polycomb transformations [7]. In humans, screening of cancer cells identified mutations of this residue in certain pediatric glioblastomas [8,9]. These oncohistone mutations are lysine-to-methionine (K27M) mis-sense substitutions that dominantly inhibit the EZH1/2 histone methyltransferases and reduce chromatin methylation [10–14]. The oncohistone is not tumorigenic on its own but may precondition cells to later oncogenic mutations [15,16]. However, since the critical window for tumorigenesis is in early developing lineages, the sequence of initiating events is not accessible to analysis.

Histones and histone modifying enzymes are conserved across eukaryotes, and expression of the H3K27M oncohistone in Drosophila cells recapitulates chromatin and silencing defects seen in gliomas [17]. Here, we use Drosophila to show that the H3K27M oncohistone blocks direct reprogramming induced by ectopic expression of the wing master regulator transcriptional activator Vestigial (Vg). While oncohistone production on its own inhibits cell proliferation, co-production with Vg results in overgrowth of cells, and these cells retain eye identity. Chromatin profiling by CUT&Tag [18] shows widespread reduction in H3K27me3 histone modification that may cripple silencing during reprogramming and identifies changes in gene expression that may induce neoplastic growth. The effect of the H3K27M oncohistone is distinct from that of eliminating the E(z) histone methyltransferase, demonstrating that a moderate defect in chromatin silencing combined with aberrant transcription factor expression can be sufficient to induce neoplastic growth, with implications for the developmental origins of gliomas.

## Results

### The H3K27M oncohistone blocks direct reprogramming

**Fig 1:**
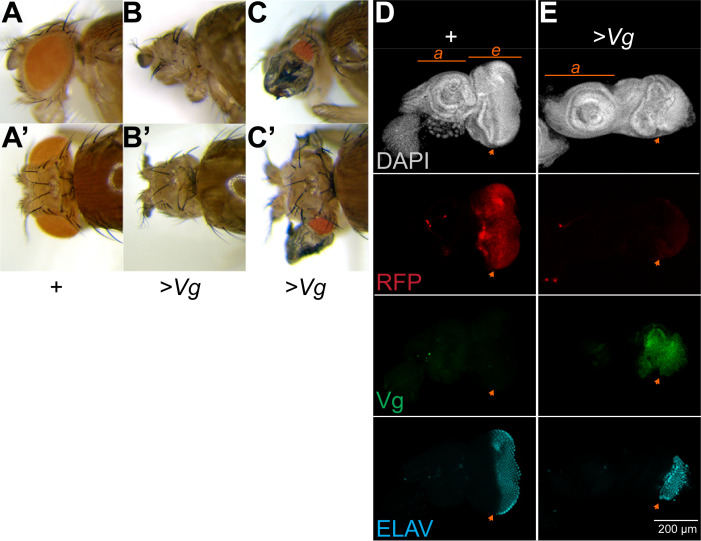
Vg expression transforms the eye into wing tissue. **(A-C)** Side and dorsal views of the head and eyes of wildtype *eyGAL/CyO* adults (**A**) and *eyGAL >Vg* adults (**B,C**). About half of all adults with *>Vg* production display pigmented wing-like projections, while the rest lack eyes entirely. **(D,E)** Eye-antennal imaginal discs from wildtype *eyGAL >RFP* larvae **(D)** and from *eyGAL >RFP >Vg* larvae **(E)**. Discs were immunostained for the Vg protein and for ELAV, a marker of differentiating photoreceptors. The antennal (a) and eye (e) portions of the disc are indicated, and the morphogenetic furrow that separates mitotically active undifferentiated cells in the anterior of the eye from differentiating photoreceptors in the posterior is marked with an orange arrowhead. The eye portions of discs with Vg production are distorted, with detectable Vg, low RFP signal, and very few differentiating photoreceptors at the posterior edge.

To probe the interaction between reprogramming and chromatin silencing, we used inducible transgenes for the Vg master regulator transcription factor and for the H3.3K27M oncohistone. Vg encodes transcriptional activation domains and–when heterodimerized with the DNA-binding Scalloped (Sd) protein–determines the identity of cells in the pouch of wing imaginal discs [19]. Vg is a master regulator of wing development, as ectopic production of Vg converts tissue into wing structures [20]. We used the *eyeless-GAL4* (*eyGAL*) driver to induce transgenes during development of the eye [21]. Expression of Vg in the eye is semi-lethal (**Table 1**), and many dying pupae have small heads that lack eye tissue (**Fig 1A and 1B**). Those adults that survive show varying degrees of transformation: some animals lack eyes, while other have reduced numbers of eye ommatidia and have darkly pigmented outgrowths resembling wing tissue with small bristles and trichome-like hairs instead of the setae of normal eyes (**Fig 1C and Table 1**). These wing-like outgrowths result from reprogramming of the eye by the Vg master regulator.

**Table 1:** Effect of Vg, H3.3 mutations, and RNAi knockdown on eye-to-wing reprogramming.

						*eyGAL4 >*					
			*H3*.*3*	*H3*.*3K27M*	*H3*.*3K27M*	*H3*.*3K27R*	*H3*.*3K9M*	*Pc_RNAi*	*Pc_RNAi*	*E(z)_RNAi*	*E(z)_RNAi*
	*+*	*Vg*	*Vg*		*Vg*	*Vg*	*Vg*		*Vg*		*Vg*
**(n)**	310	161	143	227	129	106	131	167	133	183	214
**lethal**	0	0.5	0.6	0.2	0.2	0.6	0.7	1	1	1	1
**normal eye**	1	0	0	0	0	0	0	0	0	0	0
**reduced eye**	0	0	0.04	0.8	0.02	0.04	0.02	0	0	0	0
**no eye**	0	0.05	0.12	0	0.02	0.04	0.08	0	0	0	0
**winged eye**	0	0.45	0.24	0	0	0.32	0.2	0	0	0	0
**expanded eye**	0	0	0	0	0.77	0	0	0	0	0	0

We dissected and immunostained developing eye-antennal imaginal discs to determine the cellular effects of ectopic Vg and H3.3K27M. The *eyGAL* driver induces an RFP reporter gene, marking the eye anlagen of the imaginal disc (**Fig 1D**). Inducing Vg production reduces and distorts the eye portion of discs to variable degrees, ranging from many to very few photoreceptors developing at the posterior edge of the eye disc (**Fig 1E**). While the amount of Vg protein detectable between discs varied, the RFP reporter was reduced in all discs, consistent with the inactivation of the *eyGAL* driver as Vg reprograms the eye disc.

**Fig 2:**
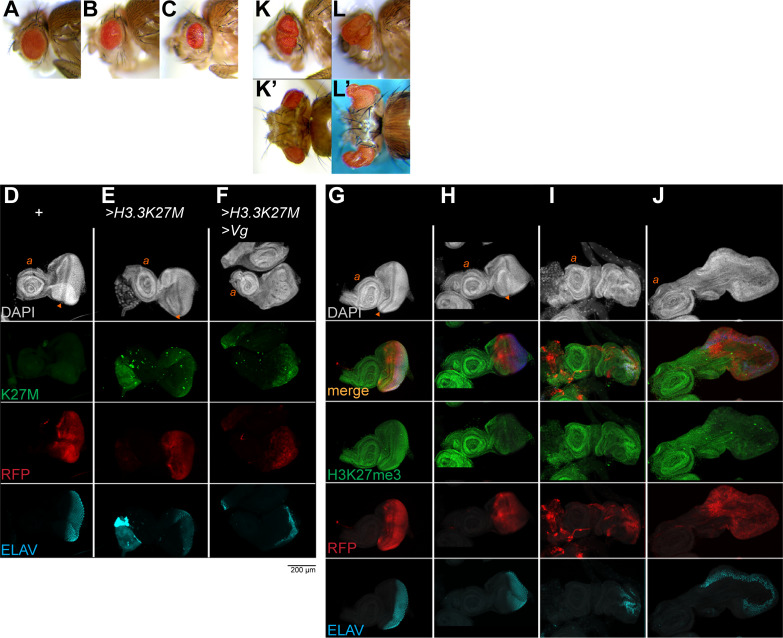
H3.3K27M oncohistones block direct reprogramming. (**A-C**) Side views of eyes of wildtype un-induced *UAS-H3*.*3K27M* adults (**A**), adults with heterozygous for *eyGAL* and *H3*.*3K27M* transgenes (**B**), or homozygous for two *eyGAL* and *H3*.*3K27M* transgenes (**C**). Production of the oncohistone reduces the size of the eye. (**D-F**) Eye-antennal imaginal discs from larvae with *eyGAL*-induced production of RFP, H3.3K27M, and Vg. Discs were immunostained for the K27M epitope and for the photoreceptor ELAV marker. The oncohistone is produced throughout the developing eye portion of the disc. (**G-J**) Eye-antennal imaginal discs with *eyGAL*-induced production of RFP, H3.3K27M, and Vg, and immunostained for the H3K27me3 histone modification and for ELAV. Histone methylation is reduced in the eye portion of the disc relative to staining in the antennal portion in discs producing the oncohistone. (**K, L**) Examples of adults with co-production of Vg and H3.3K27M in the eye. Eyes are overgrown and convoluted (**K, K’**) or overgrown with extreme projections (**L, L’**).

We used a transgene encoding an inducible H3.3K27M gene [22] and the same *eyGAL* driver to produce the oncohistone in the eye. The oncohistone decreases the size of the adult eye in proportion to dosage (**Fig 2A–2C**). Thus, while the oncohistone is associated with proliferation in cancers, on its own it inhibits tissue growth. In imaginal discs, the oncohistone is detectable throughout the eye portion of the disc (**Fig 2D and 2E**) and results in reduced staining for H3K27me3, whereas H3K27me3 staining in the antennal portion is unaffected (**Fig 2G and 2H**). These discs are slightly smaller than wildtype discs, but with normal morphology and developing photoreceptors. In contrast, the H3K27me3 modification is unaffected by ectopic expression of Vg, showing high levels in both the eye and antennal portions of discs (**Fig 2I**).

To test the importance of H3K27me3 chromatin silencing in reprogramming, we co-induced production of Vg and the K27M oncohistone together in developing eyes. Surprisingly, this completely suppressed eye-to-wing reprogramming (**Table 1**). H3.3K27M production rescues the lethality of Vg production, but the eclosing animals have grossly disrupted and expanded eyes with a variety of projections, stalks, and folds (**Fig 2K and 2L**). These convoluted eyes contain ommatidia with no wing-like tissue, implying that co-production of the oncohistone blocks the reprogramming effects of Vg, thereby maintaining the eye identity of these cells. This is apparent in developing imaginal discs, where the eye portion of discs is greatly expanded where the oncohistone is produced, with developing photoreceptors along a convoluted edge of the disc (**Fig 2F and 2J**). Suppression of reprogramming is specific to the K27M oncohistone, because a transgene with a wildtype H3.3 histone, with an H3.3K27R substitution, or with an H3K9M substitution [17] do not block reprogramming (**Table 1**). We tested additional independent *eyGAL4* driver constructs and insertions to induce the H3.3K27M oncohistone and to induce Vg (see **S1 Text**), all of which reproduced the phenotypes of Vg-induced reprogramming and oncohistone-induced overgrowth. Thus, H3K27me3-mediated silencing is required both for transcription factor-induced reprogramming of the eye and to limit factor-induced neoplastic growth.

### Vg and H3K27M induce cell death and proliferation

**Fig 3:**
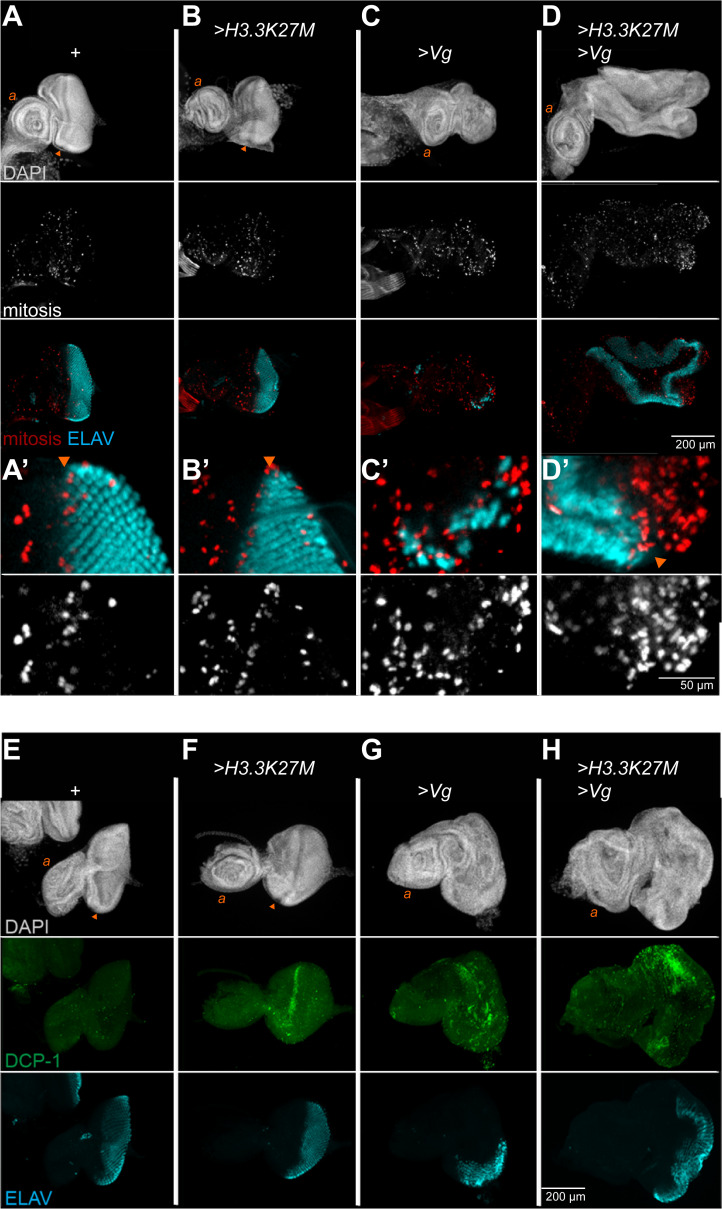
H3.3K27M and Vg stimulate cell death and proliferation. (**A-D**) Eye-antennal discs immunostained for the H3S10-phosphorylation marker of mitosis (**A-D**) with higher magnification imaging around the morphogenetic furrow (arrowheads, **A’-D’**). (**E-H**) Discs immunostained for the cleaved DCP-1 marker of apoptosis (**E-H**). The wildtype ‘+’ strain was *w*^*1118*^, and all discs were immunostained for ELAV.

The sizes of imaginal discs and adult eyes are consistent with decreased proliferation with either Vg or with H3.3K27M production, but increased proliferation with the two proteins co-produced. We examined cell division rates and cell death rates by staining eye imaginal discs with a mitotic marker histone (H3S10-phosphorylation) and with a cell death marker (cleaved DCP-1). In wildtype controls, mitoses are scattered throughout the anterior portion of the eye disc, but mostly absent in the posterior region once photoreceptors start to differentiate (**Fig 3A and 3A’**) with negligible cell death (**Fig 3E**). Expression of H3.3K27M does not affect mitosis in the disc (**Fig 3B and 3B’**), but a stripe of cell death appears across the disc where cells transition from proliferating to differentiating regions (**Fig 3F**). Thus, the reduction in eye size with H3.3K27M production is at least in part due to reduced cell viability. In contrast, induction of Vg is associated with increased mitosis and increased cell death throughout the disc (**Fig 3C and 3C’ and 3G**). Thus, reduced cell viability limits the size of the reprogrammed tissue. Finally, co-production of H3.3K27M and Vg results in very large discs with patches of mitosis frequently apparent (**Fig 3D and 3D’**), but these discs show extensive cell death in undifferentiated regions (**Fig 3H**). The hyperproliferation of some regions accounts for overgrowth in spite of extensive oncohistone-induced cell death in other regions.

### Gene expression in Vg-reprogrammed tissues

**Fig 4:**
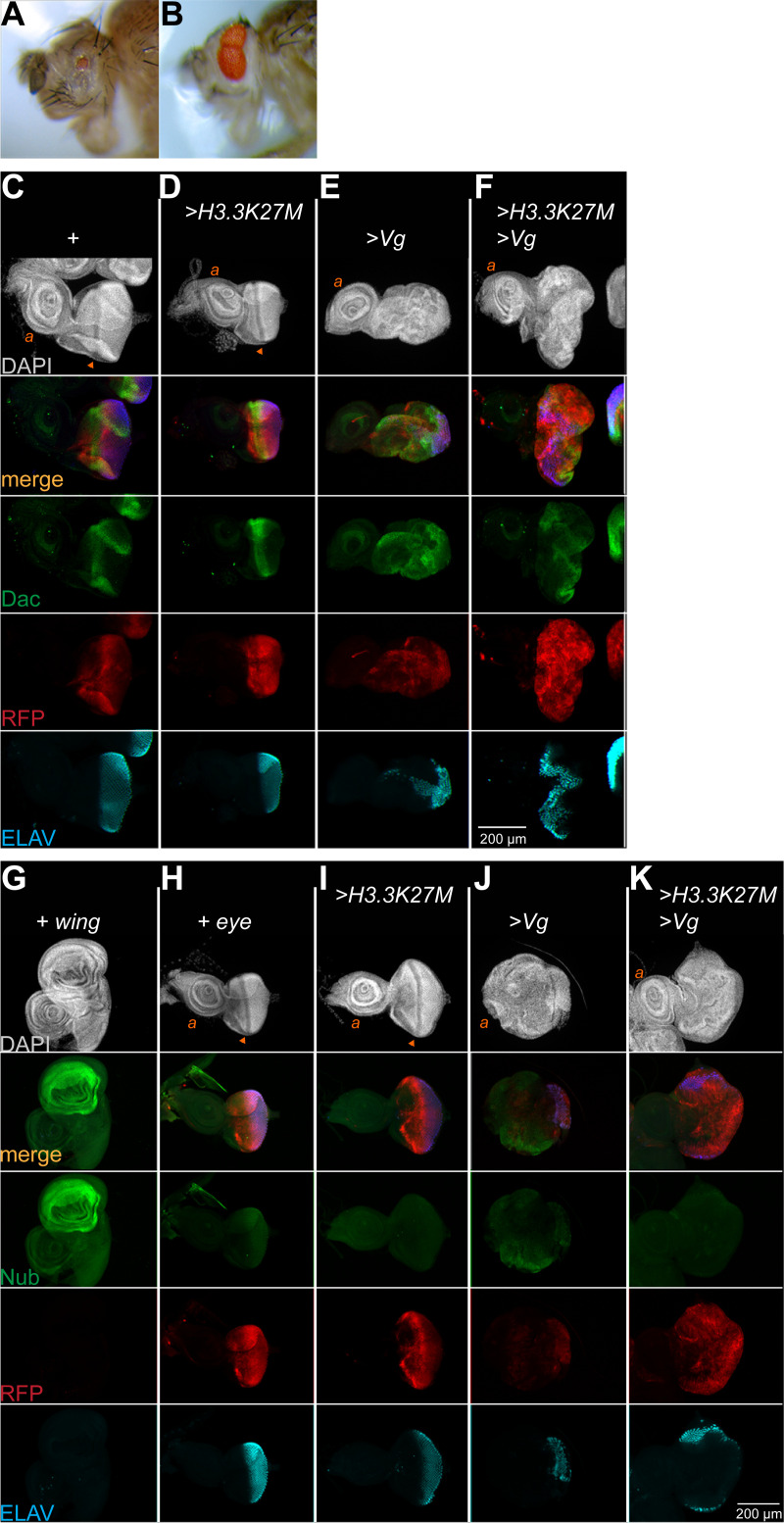
Tissue-specific gene expression changes during direct reprogramming. (**A**) Side view of a *vg*^*CL7A*^ mutant adult with *eyGAL*-induced Vg production in the eye. Eye size is reduced, but not transformed into a wing. (**B**) Side view of a *vg*^*U*^*/+* head with *eyGAL*-induced H3.3K27M and Vg co-production in the eye. The eye is convoluted but reduced in size instead of overgrown. (**C-F**) Eye-antennal discs with *eyGAL*-induced production of RFP, H3.3K27M and Vg and immunostained for the eye-specific Dac protein and for ELAV. Neither Vg nor H3.3K27M production prevent Dac expression, although these discs are disorganized. The wildtype ‘+’ strain was *eyGAL4>UASRFP/CyO*. (**G-K**) Wildtype (‘+’, *eyGAL4>UASRFP/CyO*) wing imaginal discs (**G**) and eye-antennal discs with *eyGAL*-induced production of H3.3K27M and Vg (**H-K**) immunostained for the wing-specific Nub protein and for ELAV. The wing pouch is heavily labeled with Nub, and eye discs with Vg production have patches with low level Nub staining. No Nub staining is visible in eye discs with H3.3K27M production or in discs with H3.3K27M and Vg co-produced.

Reprogramming of the eye inactivates the *eyGAL* driver once cells transform, and this is apparent by reduced RFP production when Vg is induced (**Fig 1E**). Inactivation of the *eyGAL* driver implies that the Vg transgene will also be inactivated, and so the endogenous *vg* gene must be activated for successful reprogramming. To test this, we constructed animals with the *eyGAL* driver and the inducible Vg transgene but lacking the endogenous *vg* gene. As expected, these animals have very small eyes with no wing tissue outgrowths (**Fig 4A**). This implies that transient production of Vg activates endogenous genes for wing specification. The endogenous *vg* gene is also required for overgrowth of eye discs with oncohistone and ectopic Vg expression, as a dominant negative *vg*^*U*^ allele reduces eye size (**Fig 4B**).

To characterize activation of wing-specific genes further, we stained discs for proteins specific for eyes or for wings. These discs continue to express the eye factor Dachshund (Dac), although the normal striped pattern of this factor is distorted by the gross disorganization of the reprogrammed disc (**Fig 4C–4F**). The wing specification factor Nubbin (Nub) in reprogrammed eye discs is detectable but weaker than in wing discs (**Fig 4G–4I**), implying that the wing determination program is not efficiently activated. Notably, there is no detectable production of Nub in *>H3*.*3K27M >Vg* overgrown discs (**Fig 4J**), while *RFP* and *dac* are highly expressed. Thus, in this setting the ectopic production of Vg does not activate wing determination and eye factors continue to be expressed.

### Chromatin profiling of H3K27me3 domains in reprogrammed tissues

To profile chromatin domains in Vg-reprogrammed eyes, we adapted the CUT&Tag method [18] for dissected imaginal discs from Drosophila larvae. CUT&Tag works by first soaking unfixed cells with a factor-specific antibody which binds to chromatin sites, followed by decoration with a secondary antibody. Next, a protein-A-Tn5 (pA-Tn5) transpososome loaded with adapter sequences is soaked in, binding to the chromatin-bound antibodies. Activation of the tethered transpososome by adding magnesium then integrates the adapters around binding sites, and PCR enrichment and sequencing of the resulting library thus maps the targeted chromatin protein. We have previously adapted the micrococcal nuclease-based CUT&RUN method for dissected imaginal discs [22], and adjusting buffers for CUT&Tag works reliably. For CUT&Tag with tissue samples, we dissect wing or eye imaginal discs, coat them with Concanavalin A magnetic beads for handling, lightly permeabilize them with digitonin, and sequentially incubate with antibodies and then with pA-Tn5. Resulting libraries are subjected to Illumina paired-end sequencing and mapped to the Drosophila dm6 genome assembly. Imaginal discs from 2–3 larvae were sufficient to generate chromatin profiles, although the capacity of CUT&Tag to profile very small sample sizes should work with even less tissue [17,23].

**Fig 5:**
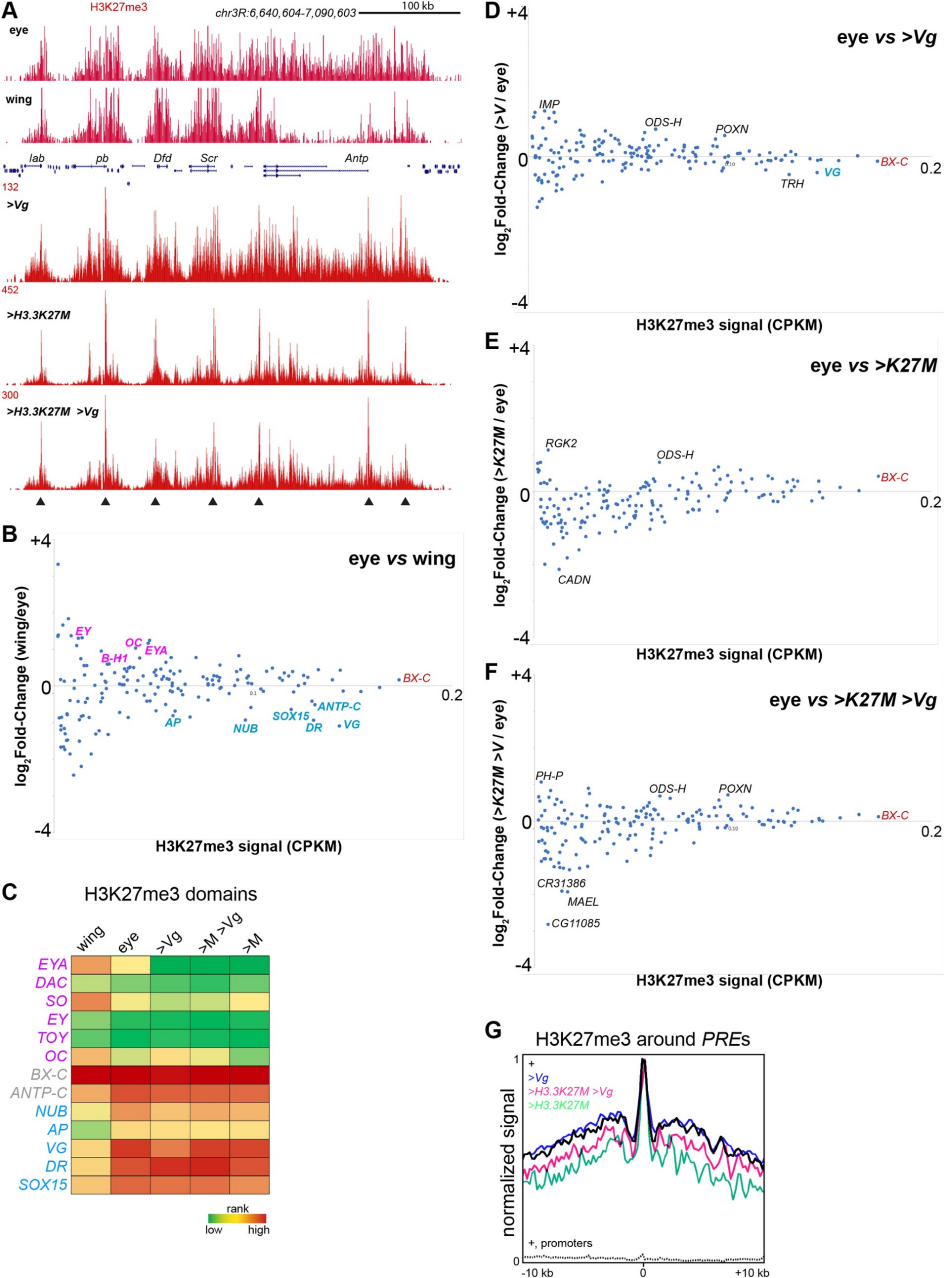
Chromatin profiling of H3K27me3 domains in reprogrammed eye imaginal discs. (**A**) Chromatin profiling at the *ANTP-C* H3K27me3 domain in wing and eye imaginal discs. Arrowheads mark the positions of major Polycomb-bound *PRE*s. (**B**) Changes in average fragment density (CPKM) of H3K27me3 in 166 annotated Polycomb domains between wing and eye imaginal discs. Domains are ranked by decreasing average H3K27me3 CPKM in wing and eye imaginal discs. Selected domains containing eye-specific genes (pink), wing-specific genes (blue) or common repressed genes (red) are marked. (**C**) Selected domains including eye-specific genes (pink), wing-specific genes (blue) or common domains (grey) are shown for wing and eye controls and production of Vg and H3.3K27M. (**D-F**) Changes in average fragment density (CPKM) of H3K27me3 in Polycomb domains between *w*^*1118*^ eye imaginal discs and discs with H3.3K27M and Vg production. Selected domains with the largest changes in chromatin methylation are marked. (**G**) Average H3K27me3 coverage around 700 Polycomb-bound *PRE*s [24] within H3K27me3 domains (solid lines), and around promoters (dashed line).

We first mapped the H3K27me3 silencing modification in eye and wing imaginal discs from wildtype larvae (**S1 Text and S1A Fig**). Previous studies found that H3K27me3-marked domains are shared between larval tissues but with quantitative differences in chromatin methylation that correspond to expression of included genes [22,24]. For example, a 350 kb H3K27me3 domain encompasses the *ANTENNAPEDIA-COMPLEX* (*ANTP-C*) cluster of homeobox genes in both eye and wing imaginal disc cells (**Fig 5A**). In eye imaginal discs the *Antp* gene is silenced and heavily coated with H3K27me3-marked chromatin, while in wing imaginal discs the *Antp* gene is transcribed, and H3K27me3 across this gene is correspondingly depleted. To quantify changes in chromatin landscapes, we measured the average H3K27me3 signal across 166 annotated H3K27me3 domains (**S1 Data**) in wing and eye discs and compared these on a scatter plot (**Fig 5B**). Domains that encompass genes involved in wing specification have moderately high chromatin methylation in eye discs and lose signal in wing discs when the genes are active. The opposite trend occurs for eye specification genes. Thus, as expected, activation of tissue-specific genes in H3K27me3 domains is accompanied by the reduction of histone methylation.

For discs producing Vg, H3.3K27M, or co-producing Vg and H3.3K27M, we dissected away the antennal portion of the disc and profiled only eye tissue. Production of Vg in eye discs results in limited changes in H3K27me3 across domains compared to eye discs (**Fig 5C and 5D** and **S1 Data**). The domains including the *vg* and the *nub* genes are noticeably less methylated but retain more methylation than in wing imaginal discs. Thus, reprogramming of eye cells is not accompanied by widespread changes in chromatin methylation patterns, and the more limited changes at wing-specific genes than in wing discs suggests that Vg expression does not fully rewire gene expression programs.

We next looked at the effect of the H3.3K27M oncohistone on H3K27me3 patterns in the eye. The oncohistone is incorporated into chromatin by histone H3.3-specific chaperones at active promoters and enhancers, and at lower levels by DNA replication throughout the genome [25], and production of H3.3K27M in eye discs gives a similar promoter enrichment with background throughout the genome when profiled using an antibody to the K27M epitope (**S1B Fig**). The oncohistone reduces H3K27me3 staining in the eye (**Fig 2D–2F**), and quantitative chromatin profiling of glioma cell lines has previously shown that H3K27me3 is globally reduced but a few domains remain [25]. In the eye imaginal disc, H3K27me3 still coats domains when the oncohistone is produced, although domains with less methylation are more depleted with more dispersion than strong domains (**Fig 5E**). Moderate depletion at weak domains is also apparent when the oncohistone and Vg are co-produced (**Fig 5F**). Additionally, we noted that the domain including the *vg* gene retains methylation, implying the endogenous gene is not being efficiently activated, and H3K27me3 signal at other wing and eye determination genes are not significantly altered (**S1 Data**).

We attributed the effects on weak domains to the overall lower H3K27me3 signal in oncohistone-producing cells. However, even in strong domains H3K27me3 distribution is affected. In Drosophila H3K27me3 domains are nucleated at nucleosome-depleted *Polycomb Response Elements* (*PRE*s), and chromatin methylation then spreads out across the domain [26]. The H3.3K27M oncohistone specifically interferes with methylation spreading [27,28], and changes in the pattern of H3K27me3 methylation is apparent at individual domains such as the *ANTP-C* (**Fig 5A**). In this domain *PRE*s are still heavily marked with the histone modification but intervals between *PRE*s are reduced, consistent with the oncohistone inhibiting spreading of the modification from *PRE*s. To assess spreading, we used published sites of Polycomb binding within H3K27me3 domains ([24] and **S1 Data**] to define the positions of *PRE*s and display H3K27me3 signals around those sites. This analysis confirmed a consistent loss of methylation around *PRE* peaks (**Fig 5E and 5G**). H3K27me3 profiling of eye disc portions co-producing Vg and H3.3K27M recapitulate reduced spreading around *PRE*s in strong domains while weak domains are more dispersed (**Fig 5E**). Thus, oncohistone production in the eye does not ablate H3K27me3 domains, but does reduce the density of methylation across all domains.

### Transcriptome profiling of reprogrammed tissues

**Fig 6:**
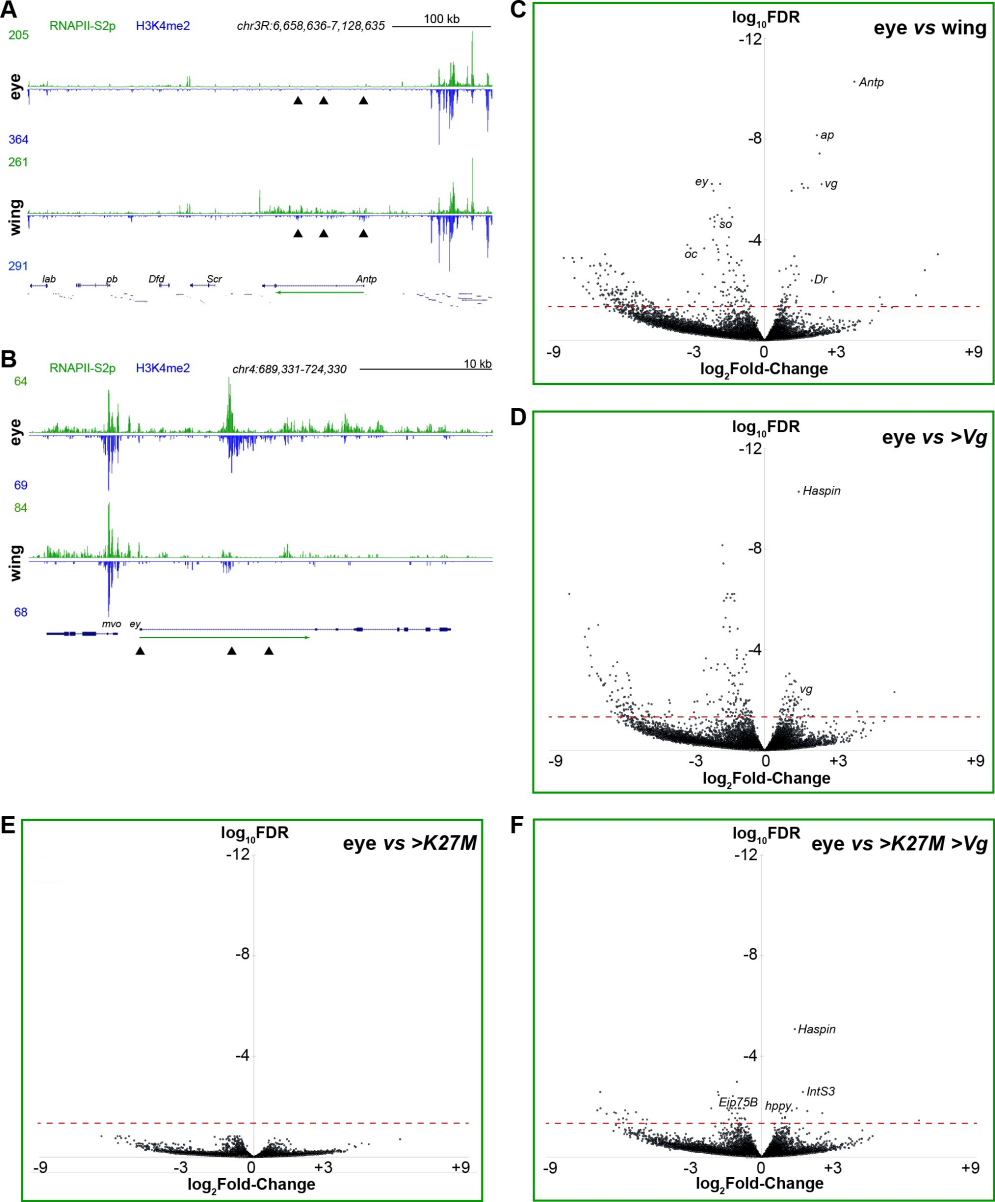
Chromatin profiling of elongating RNAPII and H3K4me2 in reprogrammed eye imaginal discs. (**A,B**) RNAPII-S2p and H3K4me2 signal across the *ANTP-C* (**A**) and the *ey* gene (**B**). Arrowheads indicate alternative promoters of the *Antp* and the *ey* genes, and the green arrow indicates the direction of transcription. (**C-F**) Volcano plots of differentially-expressed genes between wildtype (*w*^*1118*^) eye imaginal discs, wing imaginal discs, and eye discs with H3.3K27M and Vg production. Selected genes that are significantly differentially-expressed are marked.

We profiled wing and eye imaginal discs for the histone H3-K4 dimethylation (H3K4me2) histone modification to identify active promoters, and for the phosphorylated elongating form of RNA polymerase II (RNAPII-S2p) to measure transcription in each tissue. While RNA-seq profiles mature mRNA abundance in a cell, histone modification and RNAPII measurements directly correspond to changes in gene activation and silencing. Genome-wide patterns of both H3K4me2 and RNAPII-S2p are very similar between wing and eye discs, reflecting their similar expression profiles of housekeeping genes (**Fig S1B**). In contrast, wing and eye specific genes show increased promoter H3K4me2 and gene body RNAPII-S2p signals in the tissue where they are active (**Fig 6A and 6B**). Additionally, H3K4me2 signal identifies the active TSS of genes with alternative promoters (**Fig 6B**). To identify differentially expressed genes, we assigned promoter activity scores by binning H3K4me2 signal ±500 bp around each annotated promoter and assigned gene transcription scores by binning RNAPII-S2p signal across each annotated gene (**S1 Data**). Since RNAPII profiling integrates signal over the gene, we focused on this as a robust measure of gene transcription. We performed 4–6 biological replicates for each tissue with up to 10 million mapped reads per replicate and compared normalized count tables to discriminate genes in wing and in eye discs (**Fig 6C and S1 Data**). With a stringent threshold (FDR≤0.05), we identified 50 genes up-regulated in wing imaginal discs, including 15 transcription factors such as *vg*, *Antp*, *ap*, *Dr*, *nub*, *Sox15*, *rn*, and *zfh2* (**S1 Data and Fig 6C**). In contrast, 233 genes are up-regulated in eye imaginal discs, including many eye-specific transcription factors such as *Optix*, *dac*, *dan*, *eya*, *lz*, *lab*, *so*, *B-H2*, *ey*, *toy*, *gl*, and *oc*. The number of recovered differential tissue-specific transcription factors genes is slightly better than that in published RNA-seq data between eye and wing imaginal discs [29], demonstrating that RNAPII profiling efficiently detects expression changes in tissue specification genes.

We then profiled RNAPII in dissected eye discs producing Vg. There are 30 genes up-regulated in Vg-producing samples, while 138 genes are down-regulated compared to wildtype eye imaginal discs (**S1 Data and Fig 6D**). While the promoter of the endogenous *vg* gene gains H3K4me2 and is transcribed, only 51 (30%) of differentially-expressed genes are shared with wing disc samples. This may be due to the mosaicism of Vg-expressing samples or to incomplete activation of wing specification genes, but the more limited changes indicate that reprogramming of the eye by ectopic Vg is incomplete.

We then profiled RNAPII in eye discs producing the H3.3K27M oncohistone. Production of the oncohistone has little effect on genome-wide transcription (**Fig 6E and S1 Data**), consistent with the largely normal development of this genotype. Profiling of samples co-producing H3.3K27M and Vg discriminated 14 up-regulated genes and 45 down-regulated genes (**Fig 6F and S1 Data**). Notably, the endogenous *vg* gene is not induced, and only 28 differentially transcribed genes are similarly regulated in wing disc samples, consistent with the failure of reprogramming in these samples. There are 31 genes uniquely differentially transcribed in H3.3K27M and Vg co-producing discs (**S1 Data**). Of the 11 over-transcribed genes in this sample, two genes (*hppy* and *Stlk*) encode signaling kinases in the STE20 subfamily: the *happyhour* (*hppy*) kinase in particular has been linked to positive regulation of multiple developmental signaling pathways, including JNK, Egfr, hippo, TORC1, and TOR pathways [30–33], and influences both cell division and apoptosis in imaginal discs [32]. A third over-transcribed gene is *Rnf146*, which affects Wnt signaling. It is striking that human pediatric tumors with H3.3K27M mutations are associated with up-regulation of developmental signaling receptor PDGFRA [34].

Down-regulated genes might also lead to overgrowth; of 18 genes uniquely down-regulated in H3.3K27M- and Vg- co-expressing discs, *Eip78C* is part of the ecdysone-response program, while other like the transcription factor *pre-lola-G* have no known function. Finally, certain common changes in gene regulation may drive neoplastic growth of H3.3K27M- and Vg- co-expressing tissues. Two intriguing up-regulated genes are *IntS3*, a component of the general transcriptional Integrator complex [35], and *Haspin*, which encodes a mitotic histone kinase that also influences Polycomb-mediated silencing [36,37]. Further studies will be required to identify which of these genes drives neoplastic growth of eye discs co-regulating Vg and the H3.3K27M oncohistone.

### H3K27M and Vg co-expression imitates PRC1 mutants

**Fig 7:**
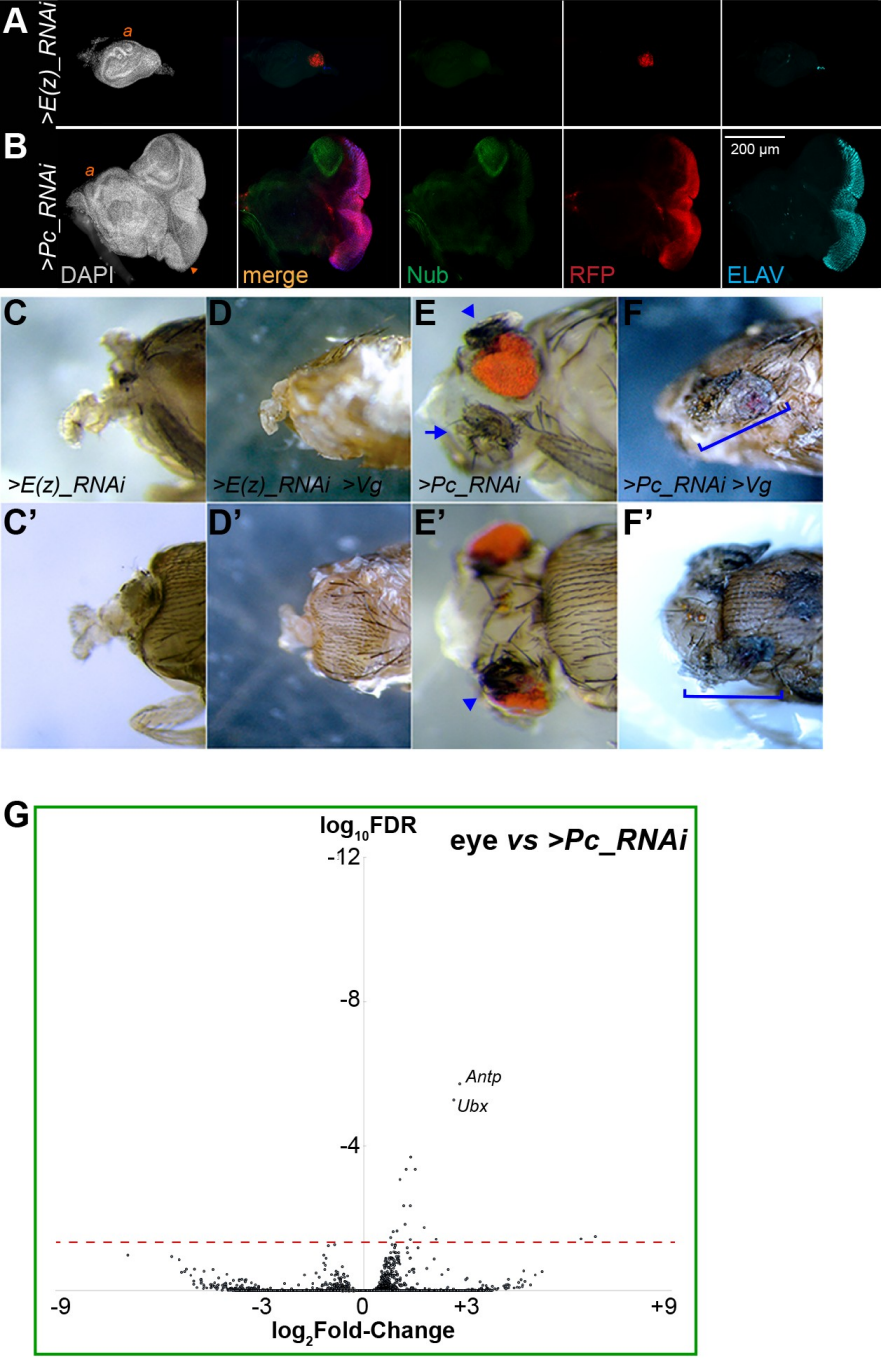
Effects of reduced PRC1 and PRC2 on direct reprogramming and cell proliferation. (**A-B**) Eye-antennal discs with *eyGAL*-induced expression of *RFP* and RNAi constructs directed against *E(z)* (**A**) or *Pc* (**B**) transcripts. Both discs are immunostained for the wing-specific Nub protein and for ELAV. The eye and antennal portions of discs are greatly reduced with *E(z)* knockdown, while *Pc* knockdown results in overgrowth of the eye portion and expression of Nub at the dorsal edge of the disc. (**C-D**) Heads of dissected dying pharate pupae with *E(z)* knockdown (**C, C’**) or *E(z)* knockdown and Vg production (**D, D’**). Loss of E(z) ablates all tissue derived from the eye-antennal disc including the head capsule and eyes, without affecting mouthparts. (**E-F**) Heads of dissected dying pharate pupae with *Pc* knockdown (**E, E’**) or *Pc* knockdown and Vg production (**F, F’**). Loss of *Pc* induces ectopic wing-like structures at the dorsal edges of eyes (arrowhead) and transforms antennae into legs (arrow). Vg production with *Pc* knockdown results greatly reduced eyes and expanded and flattened wing-like structures (bracket). (**G**) Volcano plot of differentially-expressed genes between wildtype (*w*^*1118*^) eye imaginal discs and eye discs with *Pc* knockdown. *Antp* and *Ubx* are the most significantly differentially-expressed.

Tissue reprogramming has been associated with a number of components in the Polycomb silencing system [38]. H3K27 trimethylation is catalyzed by the E(z) histone modifying enzyme, a component of the Polycomb Repressive Complex 2, while the Polycomb Repressive Complex 1 (PRC1) complex binds the H3K27me3 mark on nucleosomes [39]. PRC1 also binds many promoters independently of PRC2 or H3K27me3, implying that it has additional functions [24]. Indeed, mutation of PRC1 components result in overgrowth of imaginal discs, while mutations in PRC2 components inhibit cell growth [24,40]. We examined the roles of PRC1 and PRC2 in reprogramming of the eye. A previous study characterized *eyGAL*-induced knock-down of PRC1 and PRC2 components [41], which we repeated here. The effects of knocking down the PRC2 histone methyltransferase E(z) is much more dramatic than the expression of H3.3K27M, resulting in the near elimination of the eye portion of imaginal discs (**Fig 7A**). These animals die as pupae lacking all head structures derived from the eye-antennal imaginal disc (**Fig 7C**). Simultaneous knockdown of *E(z)* and expression of Vg similarly results in small discs and no head structures (**Fig 7D**). Thus, growth inhibition by H3.3K27M expression resembles intermediate reduction of the E(z) enzyme, and these intermediate levels separate the requirement of E(z) for reprogramming from its requirement for cell viability.

Eye-specific knockdown of *Polycomb* is also lethal, but with a phenotype distinct from *E(z)* knockdown. Previous work has also shown that *Polycomb* knockdown reprograms part of the eye to wing-like tissue and induces overgrowth in the disc [41]. Eye discs with *Polycomb* knockdown have an outgrowth on the dorsal side of the disc [41] with high expression of the wing-specific Nub transcription factor (**Fig 7B**), and the dorsal edge of the eye disc is predisposed to reprogramming [41,42]. Dying pupae have both eye and wing tissue in the head, with wing-like outgrowths out the dorsal side of the eye [41] (**Fig 7E**). Reprogramming is enhanced by simultaneous *Polycomb* knockdown and Vg expression, where the amount of eye tissue is reduced and the wing tissue expands in a more flattened structure (**Fig 7F**).

We profiled histone modifications and RNAPII from *Polycomb* knockdown discs (**S1 Data**). These discs are mosaic for eye and reprogrammed fates, nevertheless induction of some genes are detected, including *Antp* and *Ubx* (**Fig 7G and S1 Data**). This matches transformation of tissues: *Antp* is activated when eye cells are reprogrammed to a wing fate [41], and *Ubx* expression is probably due to transformation of the antennal portion of the disc to a leg fate [**Fig 7E**]. We did not expect antennal effects since the *eyGAL4* driver is limited to the eye portion of the disc in late larval stages, but this driver does transiently express in the antennal portion in earlier stages [43]. This early expression accounts for the changes in the antennal portion of discs upon *E(z)* or *Polycomb* knockdown ([41]; **Fig 7A and 7B**). Finally, immunostaining of reprogrammed eye discs detected additional induction of wing-specification genes (**Fig 7B** and [41]) which were not detected by RNAPII profiling, possibly because of low expression of these transcription factors or because of the heterogeneity of discs limits detection.

While there are similarities between our results and published effects of PRC1 and PRC2 mutations, there are key differences in the regulatory consequences when reprogramming occurs. When *eyGAL4*-induced Vg expression reprograms the eye, the *eyGAL4* driver shuts off. Similarly, successful reprogramming by *Polycomb* knockdown inactivates the *eyGAL4* driver (**Fig 7B**), and therefore *Polycomb* expression will be restored in reprogrammed cells. This is distinct from cells co-expressing H3.3K27M and Vg, or from genetic mutants of PRC1 or PRC2 [24,40]. In these cases cells must proliferate with crippled silencing. In other words, transient loss of *Polycomb* results in reprogramming, while permanent loss results in neoplastic growth. Partial reduction of E(z) activity allows cells to live, and thus reveals its requirement for reprogramming by a single transcription factor.

## Discussion

The idea that cell fate programs are reinforced by chromatin silencing of alternative pathways implies that reducing epigenetic barriers that restrict cell fates will stimulate cell fate transformations. We find the opposite–using ectopic expression of the Vg master regulator factor, we find that compromised silencing does not enhance transformation of the eye; instead cells of the eye disc hyperproliferate. It is startlingly simple to create overgrowth tumors in the Drosophila developing eye: expression of only two proteins–the H3.3K27M oncohistone and a transcription factor–are sufficient. Commitment to a developmental fate requires both the expression of genes for determinative transcription factors and the silencing of genes for alternative fates. Coordinated activation and silencing may shape and stabilize developmental trajectories. Our results highlight that chromatin silencing is essential for transcription factor-induced developmental reprogramming. Cell identity in the Drosophila eye is determined by a network of self-reinforcing transcription factors. To reprogram this tissue expression of Vg must both induce wing specification genes and silence eye-specifying factors. Surprisingly, inhibition of H3K27 methylation does not enhance reprogramming, implying that the activation of silenced wing specification genes is not limiting. Instead, our results argue that H3K27me3-mediated silencing of eye-specific factors is needed for successful reprogramming. This requirement may result from the inducing Vg in a setting where the eye determination program has already been established. Further, the differentiation of retinal cells even when Vg is expressed indicates that eye determination factors must be dominant to wing specification factors, similar to what has been observed after tissue damage in Drosophila [44]. Intriguingly, overexpression of the histone gene transcription factor Wge can also drive eye-to-wing reprogramming, but this case requires chromatin silencing mediated by the histone H3K9 methyltransferase Su(var)3-9 [29,45]. Thus, reprogramming appears to require silencing of established developmental programs by either H3K27me3- or H3K9me3-mediated pathways.

Why does inhibiting reprogramming with the H3K27M oncohistone result in neoplastic growth? Genetic studies in mammalian systems have demonstrated that H3K27M oncohistones are not sufficient to induce tumorigenesis on their own [46]. Instead, secondary mutations are necessary, and the low mutational burdens of H3K27M-bearing cancers in patients implies that specific mutations are sufficient for malignancy. Some mutations are in classical tumor suppressor genes like *TP53*, thereby *e*nhancing malignancy. In pediatric midline gliomas, additional activating mutations are often in specific signaling receptors such as *PDGFRA* or *ACVR1* [8,34,47–49], and the hyperactivation of developmental signaling probably induces the mis-expression of developmental transcription factors. Co-expression of the H3.3K27M oncohistone and Vg appears to imitate this effect, perhaps through the increased expression of the STE20-like kinases we identified by transcriptome profiling. The Vg factor both promotes a wing cell fate and stimulates cell growth [50], thus suppressing reprogramming may result in unrestrained proliferation. While mutation of the four Vg homologs in humans is not observed in gliomas, mis-expression of three paralogs are associated with other cancers [51]. More generally, the fact that simply mis-expressing a developmental transcription factor and an oncohistone stimulates proliferation suggests that other transcription factors might also do so, which would explain the cell-type- and stage-specificity of oncohistone-driven cancers.

It is striking that mutations in PRC1 components stimulate uncontrolled proliferation, while mutation of PRC2 components do not [24,40]. These two complexes normally work together to silence domains in genomes, but PRC1 also regulates gene expression of some developmental targets [52,53]. Our results suggest that mutations in PRC1 components both lose chromatin silencing and mis-express a developmental regulator, driving cell proliferation.

## Methods

### Fly strains

All crosses were performed at 25°C. All mutations and chromosomal rearrangements used here are described in Flybase (http://www.flybase.org) and sources are listed in the **S1 Text.** The *eyGAL4*-3-8 driver was used for all experiments shown here, although identical results were obtained with other *eyGAL4* constructs and insertions.

### Transgenes

Inducible H3.3 constructs were constructed by cloning the *UASp* promoter and the *His3*.*3A* ORF into the pKC27mw transformation vector [54] (a gift from L. Ringrose, Humboldt-Univerität zu Berlin), and then used site-directed mutagenesis PCR to make the K27M and K27R mutated versions. Each plasmid was injected into *y* M[*vas-int*.*Dm*]ZH-2A *w*; *P*[*attP*,*y+*,*w3’*]VIE-260B embryos by Bestgene Inc (Chino Hills, CA). This line contains two landing sites [55]; integrants at the 25C landing site were used in this study.

### Imaging imaginal discs

Imaginal discs from late 3rd instar larvae were dissected and fixed for 10 minutes in 4% formaldehyde/PBST (PBS with 0.1% triton-X100), and then incubated twice in 0.3% sodium deoxycholate/PBST for 20’ each. Samples were incubated with primary antiserum diluted in PBST supplemented with 10% goat serum at 4° overnight, and finally with fluorescently labeled secondary antibodies (1:200 dilution, Jackson ImmunoResearch). All tissues were stained with 0.5 μg/mL DAPI/PBS, mounted in 80% glycerol on slides, and imaged by epifluorescence on an EVOS FL Auto 2 inverted microscope (Thermo Fisher Scientific) with a 10X objective. At least 20 discs were imaged for each genotype. Pseudo-colored images were adjusted and composited in Adobe Photoshop and Adobe Illustrator. All antibodies used are listed in the **S1 Text.**

### Imaging adult eyes

Adults were euthanized in a freezer and then imaged using a Sony digital camera mounted on a Nikon SMZ1500 stereomicroscope. Images were color-corrected with Adobe Photoshop and composited in Adobe Illustrator.

### Chromatin profiling and sequencing

We dissected imaginal discs from 3rd instar larvae in Wash+ buffer (20 mM HEPES pH 7.5, 150 mM NaCl, 0.5 mM spermidine with Roche cOmplete protease inhibitor). We used 4 wing imaginal discs and 6 eye-antennal imaginal discs for each chromatin profiling experiment. Experiments were performed in duplicate and in parallel to minimize technical variation. We used immuno-tethered CUT&Tag chromatin profiling [18] with antibodies to histone H3K27me3 (C36B11, Cell Signalling Technology) and to histone H3K4me2 (13–0027, Epicypher) modifications. To adapt CUT&Tag for whole tissues, we coated imaginal discs with BioMag Plus Concanavalin-A-conjugated magnetic beads (ConA beads, Polysciences, Inc) in 8-tube PCR strips, and exchanged solutions on a magnetic stand (MSR812, Permagen). Tissues were incubated with primary antibody in dbe+ buffer (20 mM HEPES pH 7.5, 150 mM NaCl, 0.5 mM spermidine, 2 mM EDTA, 1% BSA, 0.05% digitonin with Roche cOmplete protease inhibitor) overnight at 4°, incubated with secondary antibody in dbe+ buffer for 1 hour at room temperature, and then incubated with protein-A-Tn5 loaded with adapters in 300Wash+ buffer (20 mM HEPES pH 7.5, 300 mM NaCl, 0.5 mM spermidine with Roche cOmplete protease inhibitor) for 1 hour. After one wash with 300Wash+ buffer, samples were incubated in 300Wash+ buffer supplemented with 10 mM MgCl_2_ for 1 hour at 37° to tagment chromatin. Reactions were stopped by addition of SDS to 0.16% and protease K to 0.3 mg/mL, incubated at 58° for 1 hour, and DNA was purified by phenol:chloroform extraction and ethanol precipitation.

Libraries were prepared as described [23], with 14 cycles of PCR with 10 second combined annealing and extension for enrichment of short DNA fragments. Libraries were sequenced for 25 cycles in paired-end mode on the Illumina HiSeq 2500 platform at the Fred Hutchinson Cancer Research Center Genomics Shared Resource. Paired-end reads were mapped to release r6.30 of the *D*. *melanogaster* genome obtained from FlyBase using Bowtie2, and to the *E coli* genome for spike-in normalization. A step-by-step protocol is posted: https://www.protocols.io/view/cut-tag-with-drosophila-tissues-bnx5mfq6

### Data analysis

Track screenshots were produced using the UCSC Genome browser (http://genome.ucsc.edu) [56]. We manually annotated H3K27me3 domains as enriched blocks in either wing or eye imaginal discs, or in profiling of larval brains [22], and are listed in **S1 Data**. We used EPDnew for promoter locations in dm6 genome assembly [57], and FB 2020_03 for gene annotations [58]. Analysis and display were done using deepTools v3.3.2 and bedTools v.2.29.2 in usegalaxy.org and in MS Excel. Promoter signals were extracted as fragment counts +500 bp around EPDnew annotated gene TSSs using ‘bedtools MultiCovBed’ in bedTools. Values for each dataset are provided in **S1 Data**. H3K27me3 signals in domains signals were extracted as fragment counts per kilobase per million reads (CPKM) across annotated H3K27me3 domains using ‘bedtools MultiCovBed’ in bedTools. Values for each dataset are provided in **S1 Data**. Differentially-expressed genes were defined counting fragments forCUT&Tag of elongating RNAPII-S2p across gene lengths using ‘bedtools MultiCovBed’ in bedTools. Count tables were imported into degust v. 4.1.1 (degust.erc.monash.edu), normalized with limma-voom and displayed as a volcano plot. Genes, fold-changes, FDRs are listed in **S1 Data**. For promoter heatmaps, profiling coverage was binned by 10 bp -1 kb to +2 kb around annotated TSSs of EPDnew promoters, ordered by mean signal and plotted using deepTools. The display range was adjusted to the maximum and minimum signals for each heatmap. For average H3K27me3 coverage around *PRE*s, we used 2000 called Polycomb-bound sites in eye imaginal discs [24] and selected the top 700 sites that fall within H3K27me3-marked domains. This list is provided in **S1 Data**. We extracted and displayed mean H3K27me3 signal +20 kb around these sites using the ‘computematrix’ and ‘plotHeatmap’ functions of deepTools with 10 bp binning. Plots were individually scaled to the maximum mean signal in each dataset. For rank-ordering of domains, CPKM for H3K27me3 across domains was sorted for each sample and heatmapped using three-color conditional formatting in MS Excel with Green-Yellow-Red for low-to-high values. The full list is provided in **S1 Data**.

### Detailed protocol at protocols.io

https://www.protocols.io/view/cut-tag-with-drosophila-tissues-bnx5mfq6.

## Supporting information

S1 Text Key Resources Table. (DOCX)

S1 Fig Chromatin profiling of wing and eye imaginal discs. (**A**) Spearman correlations between samples for H3K27me3, H3K4me2, and RNAPII-S2p coverage. Correlations between the datasets were generated and displayed by the ‘plotcorrelation’ function in deepTools. (**B**) Heatmaps of H3K4me2, RNAPII-S2p, and K27M coverage centered on gene starts in *>H3*.*3K27M* and *>H3*.*3K27M >Vg* eye discs. Bedgraph files for K27M profiling were used for heatmapping by the ‘computematrix’ with 1 bp binning and ‘plotHeatmap’ functions ordered by decreasing signal in deepTools.
(EPS)

S1 Data Samples and features. Sheet 1: Profiling sample IDs. Sheet 2: H3K27me3 domains coordinates and CPKM table. Sheet 3: Differentially-marked H3K27me3 domains between eye and wing, eye and >Vg, eye and >H3.3K27M, and eye and >H3.3K27M >Vg imaginal discs. Sheet 4: Called Polycomb sites curated from [24]. Sheet 5: EPDnew promoters and H3K4me2 count table. Sheet 6: Differentially-expressed genes between eye and wing imaginal discs. Sheet 7: Differentially-expressed genes between eye and *>Vg* imaginal discs. Sheet 8: Differentially-expressed genes between eye and *>H3*.*3K27M* imaginal discs. Sheet 9: Differentially-expressed genes between eye and *>H3*.*3K27M >Vg* imaginal discs. Sheet 10: Differentially-expressed genes between eye and *>Polycomb_RNAi* imaginal discs. Sheet 11: Differentially-expressed genes between eye and wing imaginal discs. Sheet 12: Differentially-expressed genes between eye and *>Vg* imaginal discs. Sheet 13: Differentially-expressed genes between eye and *>H3*.*3K27M* imaginal discs. Sheet 14: Differentially-expressed genes between eye and *>H3*.*3K27M >Vg* imaginal discs. Sheet 15: Differentially-expressed genes between eye and *>Polycomb_RNAi* imaginal discs.
(XLSX)
