# Supplementary material for: The H3.3K27M oncohistone antagonizes reprogramming in Drosophila
Source: PLoS Genet. 2021 Jul 19;17(7):e1009225. doi: 10.1371/journal.pgen.1009225 (PMC8320987; doi:10.1371/journal.pgen.1009225)
Supplement: S1 Text — (DOCX) [file pgen.1009225.s001.docx]

**S1 Text. Key Resources Table.**

**Reagent type Designation Source^*^ Reference Additional information**

Transgene UAS-vg BDSC #37296 [1]

Transgene ey-GAL4-3-8 BDSC #5534 [2]

Transgene ey-GAL4-3-5 BDSC #8221 Exelixis, Inc

Transgene ey-GAL4-4-8 BDSC #5535 [2]

Transgene en-GAL4-e16E BDSC #30557 [3]

Transgene UAS-RFP-2 BDSC #30556 [4]

Transgene UASp-H3.3 K. Ahmad this study

Transgene UASp-H3.3K27M K. Ahmad [5]

Transgene UASp-H3.3K27R K. Ahmad this study

Transgene UASp-H3.3K27M.F BDSC #58412 [6]

Transgene UASp-H3.3K9M.F BDSC #58411 [6]

Allele vg^CL7A^ K. Ahmad [5]

Inversion vg^U^ BDSC #1350 [7]

Transgene UAS-E(z).TRiP BDSC #33659 [8]

Transgene UAS-Pc.TRiP BDSC #33622 [8]

Antibody anti-H3K27me3 CST C36B11 [9]

Antibody anti-H3K27M CST D3B5T [10] rabbit, 1:100 dilution

Antibody anti-ELAV DSHB 7E8A10 [11] rat, 1:200 dilution

Antibody anti-H3S10p CST 9706 [12] mouse, 1:200 dilution

Antibody anti-Vg S. Carroll [13] rabbit, 1:50 dilution

Antibody anti-Ct DSHB 2B10 [14] mouse, 1:100 dilution

Antibody anti-Nub DSHB 2D4 [15] mouse, 1:20 dilution

Antibody anti-Dac DSHB mAbdac2-3 [16] mouse, 1:20 dilution

Antibody anti-DCP1 CST 9578 [17] rabbit, 1:100 dilution

Antibody anti-RNAPII-S2p CST13499 [18] rabbit, 1;100 dilution

Antibody anti-H3K4me2 Epicypher 13-0027 [19] rabbit, 1:100 dilution

Transpososome protein A-Tn5 S. Henikoff [20] 1:200 dilution

*BDSC, Bloomington Drosophila Stock Center, Indiana University, Bloomington IN; CST, Cell Signalling Technology, Inc.; DSHB, Developmental Studies Hybridoma Bank, University of Iowa, Iowa City IA.

**References**

1. Kim J, Sebring A, Esch JJ, Kraus ME, Vorwerk K, Magee J, Carroll SB (1996). Integration of positional signals and regulation of wing formation and identity by Drosophila vestigial gene. Nature 382:133-8. PMID: 8700202.

2. Hazelett DJ, Bourouis M, Walldorf U, Treisman JE (1998). decapentaplegic and wingless are regulated by eyes absent and eyegone and interact to direct the pattern of retinal differentiation in the eye disc. Development 125:3741-51. PMID: 9716539.

3. Harrison DA, Binari R, Nahreini TS, Gilman M, Perrimon N (1995). Activation of a Drosophila Janus kinase (JAK) causes hematopoietic neoplasia and developmental defects. EMBO J. 14:2857-65. PMID: 7796812.

4. Wen H, Andrejka L, Ashton J, Karess R, Lipsick JS (2008). Epigenetic regulation of gene expression by Drosophila Myb and E2F2-RBF via the Myb-MuvB/dREAM complex. Genes Dev. 22:601-14. doi: 10.1101/gad.1626308. PMID: 18316477.

5. Ahmad K, Spens AE (2019). Separate Polycomb Response Elements control chromatin state and activation of the vestigial gene. PLoS Genet. 15:e1007877.doi: 10.1371/journal.pgen.1007877. PMID: 31425502.

6. Herz HM, Morgan M, Gao X, Jackson J, Rickels R, Swanson SK, et al (2014). Histone H3 lysine-to-methionine mutants as a paradigm to study chromatin signaling. Science 345:1065-70. doi: 10.1126/science.1255104. PMID: 25170156.

7. Ives PT (1956). New mutants report. Drosophila Information Service 30:72-73.

8. Perkins LA, Holderbaum L, Tao R, Hu Y, Sopko R, McCall K, et al (2015). The Transgenic RNAi Project at Harvard Medical School: Resources and Validation. Genetics 201:843-52. doi: 10.1534/genetics.115.180208. PMID: 26320097.

9. Bechet D, Gielen GG, Korshunov A, Pfister SM, Rousso C, Faury D, et al (2014). Specific detection of methionine 27 mutation in histone 3 variants (H3K27M) in fixed tissue from high-grade astrocytomas. Acta Neuropathol. 128:733-41. doi: 10.1007/s00401-014-1337-4. PMID: 25200321.

10. Fons NR, Sundaram RK, Breuer GA, Peng S, McLean RL, Kalathil AN, et al (2019). PPM1D mutations silence NAPRT gene expression and confer NAMPT inhibitor sensitivity in glioma. Nat Commun. 10:3790. doi: 10.1038/s41467-019-11732-6. PMID: 31439867.

11. O’Neill EM, Rebay I, Tjian R, Rubin GM (1994). The activities of two Ets-related transcription factors required for Drosophila eye development are modulated by the Ras/MAPK pathway. Cell 78:137-47. PMID: 8033205.

12. Miyagi S, Saito T, Mizutani K, Masuyama N, Gotoh Y, Iwama A, et al (2004). The Sox-2 regulatory regions display their activities in two distinct types of multipotent stem cells. Mol Cell Biol. 24:4207-20. PMID: 15121842.

13. Williams JA, Bell JB, Carroll SB (1991). Control of Drosophila wing and haltere development by the nuclear vestigial gene product. Genes Dev. 5:2481-95. PMID: 1752439.

14. Blochlinger K, Bodmer R, Jan LY, Jan YN (1990). Patterns of expression of cut, a protein required for external sensory organ development in wild-type and cut mutant Drosophila embryos. Genes Dev.4:1322-31. PMID: 1977661.

15. Averof M, Cohen SM (1997). Evolutionary origin of insect wings from ancestral gills. Nature 385:627-30. PMID: 9024659.

16. Mardon G, Solomon NM, Rubin GM (1994). dachshund encodes a nuclear protein required for normal eye and leg development in Drosophila. Development 120:3473-86. PMID: 7821215.

17. Sarkissian T, Timmons A, Arya R, Abdelwahid E, White K (2014). Detecting apoptosis in Drosophila tissues and cells. Methods 68:89-96. doi: 10.1016/j.ymeth.2014.02.033. PMID: 24613678.

18. Pherson M, Misulovin Z, Gause M, Mihindukulasuriya K, Swain A, Dorsett D. (2017). Polycomb repressive complex 1 modifies transcription of active genes. Sci Adv. 3:e1700944. doi: 10.1126/sciadv.1700944. PMID: 28782042.

19. Shah RN, Grzybowski AT, Cornett EM, Johnstone AL, Dickson BM, Boone BA, et al (2018). Examining the Roles of H3K4 Methylation States with Systematically Characterized Antibodies. Mol Cell 72:162-177.e7. doi: 10.1016/j.molcel.2018.08.015. PMID: 30244833.

20. Kaya-Okur HS, Wu SJ, Codomo CA, Pledger ES, Bryson TD, Henikoff JG, et al (2019). CUT&Tag for efficient epigenomic profiling of small samples and single cells. Nat Commun. 10:1930. doi: 10.1038/s41467-019-09982-5. PMID: 31036827.
